# Clinical significance of the anteromedial talus osteophyte in anteromedial ankle impingement in chronic lateral ankle instability

**DOI:** 10.1186/s13018-023-03630-x

**Published:** 2023-03-01

**Authors:** Jieyuan Zhang, Xueqian Li, Shaoling Fu, Kai Yang, Zhongmin Shi

**Affiliations:** 1 Department of Orthopedic Surgery, Shanghai Sixth People’s Hospital, 600 Yishan Road, Shanghai, 200233 China; 2 Department of Radiology, Shanghai Sixth People’s Hospital, 600 Yishan Road, Shanghai, 200233 China

**Keywords:** Chronic lateral ankle instability, Anteromedial ankle impingement, Osteophyte, Arthroscopy, Visualization, Quantification

## Abstract

**Purpose:**

The aim of this study was to evaluate the relation between anteromedial ankle osteophytes (AMAO) and anteromedial ankle impingement (AMAI) in chronic lateral ankle instability (CLAI) through visualization and quantification.

**Methods:**

Forty-three patients with unilateral CLAI between September 2018 and March 2020 accepted arthroscopic repair of an anterior talofibular ligament (ATFL) and were split into two groups: AMAI (AMAI including intraoperative AMAO resection) and pure CLAI (with AMAO but without AMAI, no AMAO resection). The AMAO protrusion lengths in each direction were measured and compared after all of the ankles were reconstructed. All patients were assessed preoperatively and at 2-year follow-up with ankle dorsiflexion, the American Orthopedic Foot and Ankle Society (AOFAS) ankle–hindfoot score, and visual analog scale (VAS) score.

**Results:**

Intelligent analysis showed that a large extent of osteophytes was found at the dorsomedial surface of the talar neck in AMAI group. The upper and inner bound protrusion distances of AMAO in AMAI group were greater than in the pure CLAI group. There was no significant difference in anterior bound protrusion distance of AMAO between the two groups. Preoperatively, the ankle dorsiflexion of AMAI group (7.6 ± 1.4°) was considerably lower than that of pure CLAI group (22.4 ± 1.9°) (*p* < 0.001). When compared to the pure CLAI group, the AMAI group had a substantially worse AOFAS score (62.2 ± 6.7 vs 71.1 ± 9.1; *p* < 0.001) and VAS score (6.0 ± 1.0 vs 4.9 ± 0.8; *p* < 0.05). However, there was no significant difference in postoperative ankle dorsiflexion, AOFAS score, or VAS score between the two groups.

**Conclusion:**

AMAO is formed mostly on the dorsomedial surface of the talar neck in CLAI with AMAI, and the upper and inner bound protrusion lengths of AMAO were shown to be significantly correlated with the existence of AMAI in CLAI.

*Level of evidence IV*.

## Introduction

Ankle sprains are one of the most frequent lower-limb injuries, and the majority of them heal with no lasting effects [1]. However, over 20% of individuals suffer from chronic lateral ankle instability (CLAI) [2, 3]. The anterior talofibular ligament (ATFL), calcaneofibular ligament (CFL), and posterior talofibular ligament (PTFL) make up the lateral ankle ligament complex, which is one of the most critical structures for maintaining lateral ankle stability [4, 5]. The majority of CLAI patients who follow conservative treatment may effectively manage their instability; however, for those who do not respond to conservative treatment, lateral ankle ligament complex repair/reconstruction is frequently recommended [6]. It is worth mentioning that the ATFL is the most commonly injured ligament structure, and in some cases, the only one [7, 8]. ATFL is the key anatomical component that maintains lateral ankle stability, according to recent study, and ATFL repair/reconstruction alone is sufficient to restore lateral ankle stability [9, 10]. Since its first publication in 1987, arthroscopic ankle stabilization has shown to be a viable alternative to the open approach [11]. With advancements in ankle arthroscopy, arthroscopic lateral ankle ligament repair has proven to be a safe, reliable, and repeatable surgical method that might become the standard in the future [12, 13].

Ankle impingement is becoming more well recognized as a consequence of recurrent ankle sprains, and it is thought to be caused by CLAI. CLAI patients frequently have anterior ankle impingement with soft tissue and/or osseous anomalies [14, 15]. Anterolateral ankle impingement (ALAI) syndrome and anteromedial ankle impingement (AMAI) syndrome are currently thought to be two distinct conditions [16]. Unlike with ALAI, the specific process that causes CLAI with AMAI is unknown. Direct trauma or recurrent microtrauma between the ankle and external objects is one accepted explanation for osteophytes formation in AMAI alone [15]. Biomechanical studies have shown that this contact has the necessary force and position on the anteromedial side of the talus to produce abnormal ossification [17]. CLAI may lead to pathological changes in the ATFL, and the talar may extrude anteriorly with dorsiflexion and come into contact with the anterior tibial margin. This may result in direct or recur microtrauma between the anteromedial tibia and talus [18, 19], and the formation of anteromedial ankle osteophytes (AMAO). Recent investigations have also found that the discomfort in anterior ankle impingement is mainly caused by compressed inflammatory soft tissue along the anterior tibiotalar joint line during forceful dorsiflexion, rather than by direct contact between talar and tibial osteophytes [20, 21]. The results of arthroscopic debridement of AMAI have also been reported with satisfactory results on multiple instances [16, 22]. However, no research on the location of osteophytes that are most likely to produce AMAI or the quantification of the osteophytes is currently available.

CT scans have been frequently utilized to examine the three-dimensional morphology of the ankle in CLAI patients [23]. It can detect osteophytes and similar lesions in general, but specific information on osteophytes is unavailable. In this study, we created a novel approach of intelligent analysis AMAO for visualization and quantification by combining three-dimensional ankle reconstruction with Arigin3D-STS-Design software.

As a result, the goal of this study was to visualize the preference site of AMAO in CLAI with AMAI and quantify the link between AMAO and AMAI. The imaging and measurement of osteophytes provide a more accurate guidance for arthroscopic therapy.

## Methods

### Study design and patients

This was a retrospective study carried from September 2018 to March 2020 in Shanghai Sixth People’s Hospital, and 43 patients (25 males and 18 females; average age, 38.4 years; age range, 20–65 years), who had persistent ankle pain and associated activity restrictions despite at least 6 months of nonoperative management, mechanical instability with manual anterior drawer test (ADT) stress maneuvers, with or without recognizable tenderness on palpation medial to the tibialis anterior tendon, and follow-up for at least 2 years, were assessed for study inclusion. Preoperative plain radiography and magnetic resonance imaging (MRI) were also collected. All patients agreed to arthroscopic ATFL repair and were separated into two groups: AMAI group (AMAI including intraoperative AMAO resection; 15 males and 14 females) and pure CLAI (with AMAO but without AMAI, no AMAO resection; 10 males and 4 females). This grouping method was based on whether AMAO was resected arthroscopically and the related data were gathered by two senior surgeons at the last follow-up. For each indicator, each surgeon averaged three measurements to determine the measurement result, and the final result was the average of the two surgeons’ measurement results. Patients were excluded if they had ALAI, manifest tibiotalar osteoarthritis, severe peripheral angioneuropathy, soft tissue infection at the surgical site, or a history of mental illness. All participants provided written informed consent, and the study was approved by our institution’s Ethics Committee.

Preoperative and 2-year follow-up measurements of ankle dorsiflexion, the American Orthopedic Foot and Ankle Society (AOFAS) ankle–hindfoot score, and the visual analog scale (VAS) were taken. Ankle dorsiflexion measurement was taken with a goniometer (Xu Lang Xing, China). Participants were sitting on examination table during testing, with the ankle and foot suspended over the end of the examining table and the subtalar joint in neutral position. Participants were instructed to actively dorsiflex the ankle to an extreme. The axis of the goniometer was placed near to the lateral malleolus, the stationary arm was aligned with the head of fibula, and the movable arm was aligned parallel to the plantar aspect of the calcaneus and fifth metatarsal [24]. In all cases, the same pre- and postoperative questionnaires were used and the ankle dorsiflexion angles were measured.

### Operative procedure and rehabilitation

A tourniquet was used to prevent intraoperative bleeding after general anesthesia was administered. Traditional anteromedial and anterolateral portals are used for diagnostic ankle arthroscopy. This arthroscopy should include the removal of pathologically hypertrophied synovium and fibrous tissue such as the lateral malleolus, lateral gutter, anterior and inferior of the anterior lower tibiofibular ligament, anterior distal of the tibia, tibiotalar space, talar neck, medial malleolus, and medial gutter, as well as the arthroscopic treatment of other pathologic conditions such as talus osteochondral lesions. Furthermore, the patients in the AMAO group had AMAO resection, and the resection range is appropriate for ankle dorsiflexion and inversion without impingement under arthroscopic visualization until the medial gutter of the ankle mortise is exposed. Then, double suture anchors (Smith & Nephew, Andover, MA) with a diameter of 2.9 mm are placed to the adequate visualization of the anterior distal face of the fibula. The first bone anchor is placed 1 cm superior to the tip of the fibula, and the second bone anchor is then placed 1 cm superior to the first anchor in the anterior face of the fibula. The ATFL is repaired with the arthroscopic modified Broström technique [25]. The ankle is held in neutral to slight eversion, and then, the sutures are tightened and knotted. Following complete arthroscopy, the portals were closed using a single nylon suture and the ankle is tested for stability.

The affected extremity was wrapped with and elastic bandage and elevated, local ice compress for 24 h. The suture was removed 2 weeks postoperatively. The toes, hips, and knees could be exercised on the first day postoperatively, as well as slight dorsiflexion and plantar flexion of the ankle. Patients used crutches and followed partial weight bearing with walking boots for 4 weeks. After that, patients began to abandon the crutches and started weight-bearing activities with walking boots. Until 6 weeks postoperatively, patients began normal weight-bearing activities without walking boots. Noteworthy, passive varus and valgus of the ankle were forbidden within 6 weeks postoperatively. In the follow-up regularly, we performed physical examinations and clinical evaluations of the patients and guide further functional exercise.

### Ankle model reconstruction and intelligent analysis

CT scans (64 detector rows, 0.625-mm-thick slices, 120 kV, 125 mA) of each patient’s affected side ankle were performed before and after arthroscopic surgery, and the resulting Digital Imaging and Communications in Medicine (DICOM) data were used. Arigin 3D Pro (Shanghai Xinjian Medical Technology Co.) was used to analyze DICOM images and create a three-dimensional reconstruction of the ankle. To achieve the desired effect, the software operation interface adjusts the window width and window level. Filtering and noise reduction are used in two-dimensional image preprocessing. Different gray thresholds were set in the cross section, coronal plane, and sagittal plane to distinguish bone from soft tissue.

Artificial intelligence in the Arigin3D-STS-Design software identified osteophytes, and the clipping algorithm was used to remove the part of the surface distance of less than 1 mm. The parameters of the osteophytes were then measured using the Arigin3D-STS-Design measurement module. The postoperative model was then superimposed onto the preoperative model using the ICP (iterative closest point) algorithm. Finally, we used a proximity mapping technique to quantify the morphologic changes in the postoperative model compared to the preoperative model.

### Statistical analysis

The statistical software SPSS Version 19.0 (IBM, Chicago, IL, USA) was used for the analysis. In order to create normal curves and P-P plots for normality testing, the dorsal protruding distance, medial convex distance, and anterior convex distance of osteophytes were calculated using frequency distribution. If they adhered to the normal distribution, they were described by x ± s, and the t test of two independent samples was used to compare the two groups. Otherwise, for data that did not conform to normal distribution, a two-sample Wilcoxon rank-sum test was used to compare continuous variables. *P* values less than 0.05 were considered statistically significant.

## Results

**Fig. 1:**
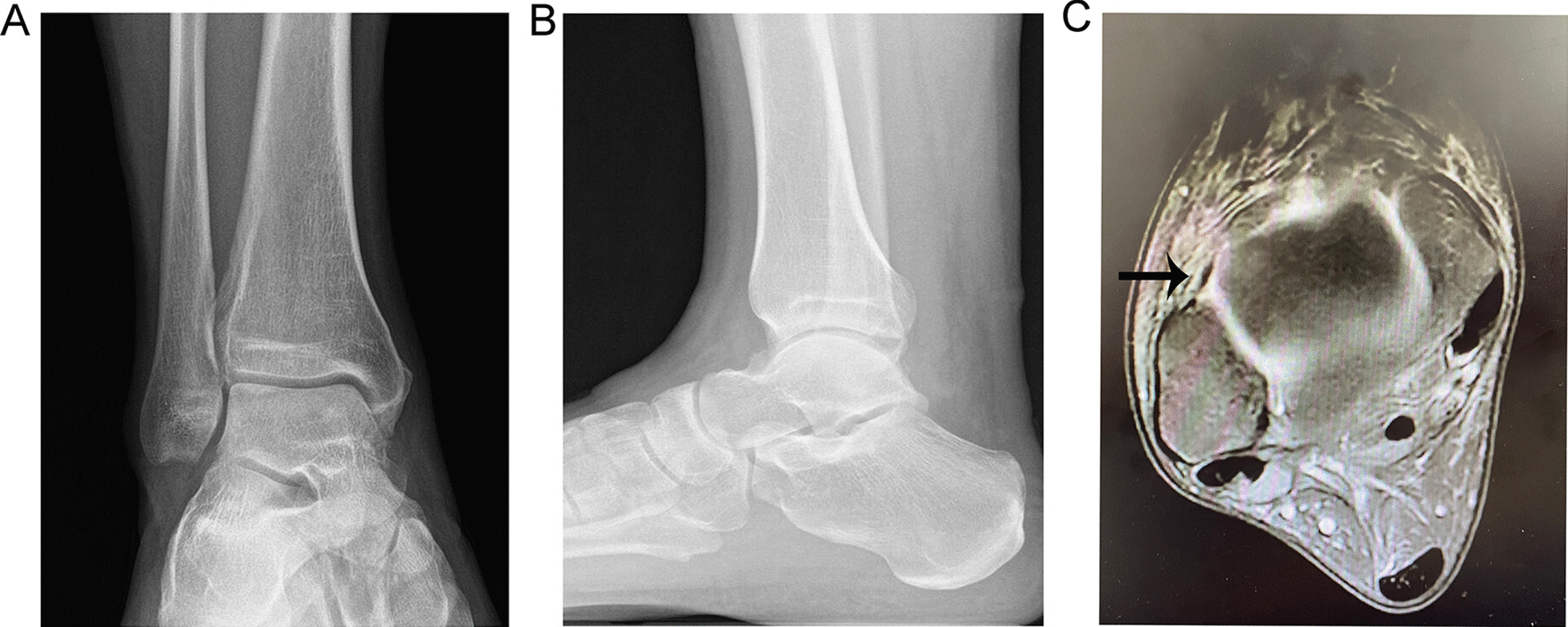
Preoperative imaging assessment of the ankles in the AMAI group by plain radiograph and MRI. The anteroposterior (**a**) and lateral (**b**) plain radiographs of the ankle. **c** Axial MRI image showing a tear of the ATFL (arrow)

**Fig. 2:**
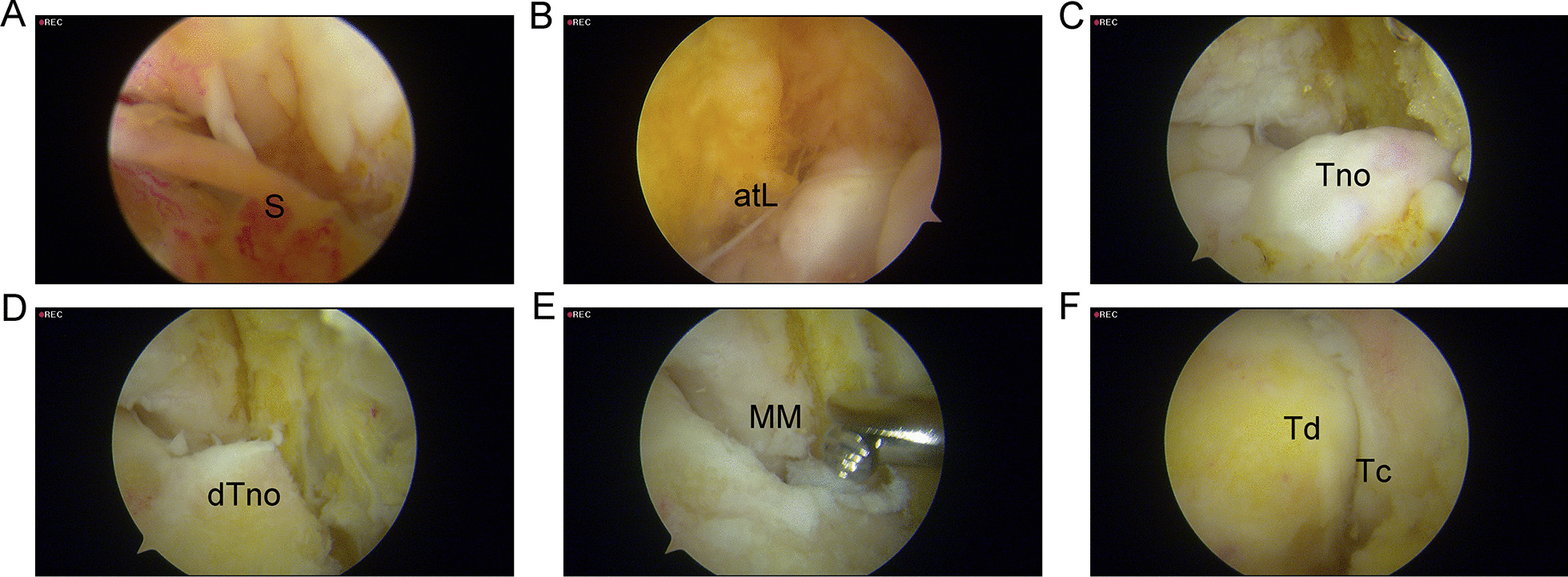
Arthroscopic images of AMAI group. **a** Arthroscopic image of AMAI before arthroscopic resection; the soft tissue synovial hyperplasia can be noted. **b** ATFL injury. **c** Talar neck osteophytes can be seen after inflamed synovium and any soft tissue clearance. **d** The medial sulcus of the ankle mortise can be exposed after dorsomedial talar neck osteophytes resection. **e** The resector can be used to remove anteromedial osteophytes. **f** There is no impingement after ankle mortise cleaning. S, synovial hyperplasia; atL, anterior talofibular ligament; Tno, Talus neck osteophyte; dTno, dorsomedial talar neck osteophytes; MM, medial malleolus; Tc, tibial cartilage; and Td, talar dome

Preoperative imaging (plain radiographs and MRI) was used to diagnose CLAI with AMAI (Fig. 1). The chronologies of injury were 45.2 ± 12.3 months in AMAI group and 39.4 ± 10.9 months in Pure CLAI group, and there was no statistically significant difference between the two groups. All 43 patients had arthroscopic surgery, which took 30–45 min in the pure CLAI group and 50–70 min in the AMAO group. There were no obvious osteophytes impingement in ankle dorsiflexion and plantarflexion after resection in the AMAI group (Fig. 2). All patients had no early complications such as wound infection, necrosis, or neurovascular injury, and all surgical incisions healed in the I stage postoperatively. At the 2-year follow-up, no patient complained of ankle instability, and the ADT of all ankles was found to be negative. It is worth noting that no AMAI occurred in the patients of the pure CLAI group at the 2-year follow-up.

### Clinical outcomes

The AMAI group’s ankle dorsiflexion was considerably lower than the pure CLAI group’s before surgery. The AMAI group’s ankle dorsiflexion improved significantly after surgery, whereas the pure CLAI group’s ankle dorsiflexion did not change appreciably (Table 1). When compared to the pure CLAI group, the AMAI group had a considerably worse AOFAS and VAS score preoperatively. The AMAI group’s functional scores improved dramatically after surgery. There was no significant difference in ankle dorsiflexion, AOFAS score, or VAS score between the two groups at the 2-year follow-up (Table 1).

**Table 1:** Ankle dorsiflexion and functional scores between the AMAI group and the pure CLAI group

Variable		AMAI group (*n* = 29)	Pure CLAI group (*n* = 14)	*p* value
Ankle dorsiflexion (°)	Pre-	7.6 ± 1.4	22.4 ± 1.9	< 0.001
	2-year-	21.3 ± 2.1	22 ± 1.8	n.s
AOFAS score	Pre-	62.2 ± 6.7	71.1 ± 9.1	0.0008
	2-year-	93.7 ± 7.8	94.2 ± 6.5	n.s
VAS	Pre-	6.0 ± 1.0	4.9 ± 0.8	0.0010
	2-year-	1.8 ± 1.3	1.7 ± 1.5	n.s

*AMAI* anteromedial ankle impingement, *CLAI* chronic lateral ankle instability; AOFAS, American Orthopedic Foot and Ankle Society, *VAS* Visual Analogue Scale, *Pre-* preoperatively, *2-year-* 2-year follow-up

“*p* value” indicated the comparison between the AMAI group and the pure CLAI group

### Osteophytes visualization and quantification

**Fig. 3:**
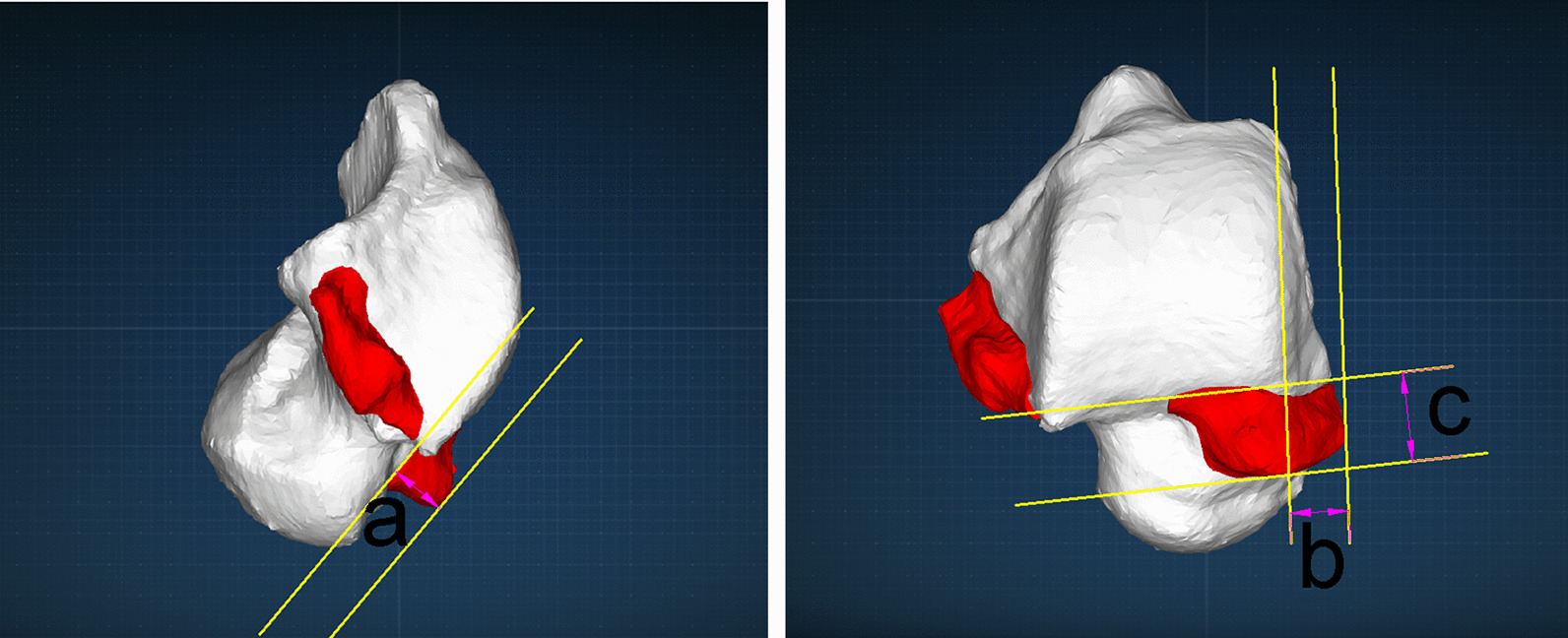
The preoperative quantitative examination of talar neck osteophytes. After the Arigin3D-STS-Design software intelligently identified the osteophytes, the upper (**a**), inner (**b**), and anterior (**c**) bound protrusion distances of the dorsomedial talar neck osteophytes were measured

**Fig. 4:**
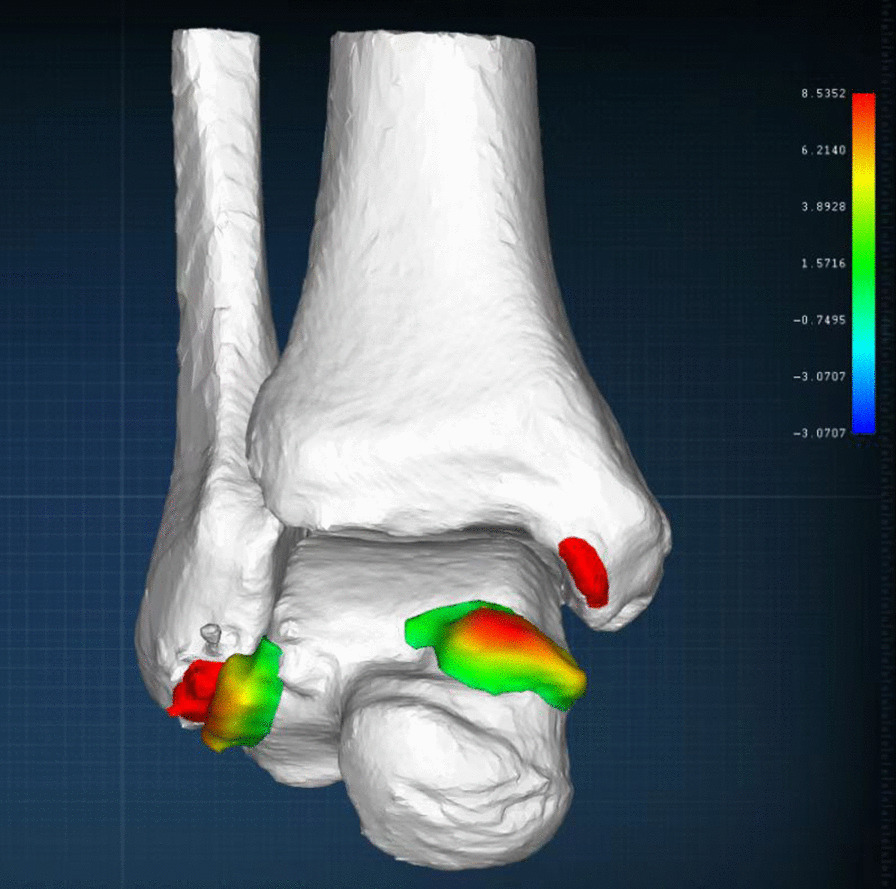
Visualization of the morphologic changes in the postoperative model relative to the preoperative model. The postoperative model was superimposed onto the preoperative model. The red and blue colors were deviations of the preoperative model outside and inside of the postoperative model

Preoperatively, intelligent identification using the Arigin3D-STS-Design software revealed that the AMAI group had a large extent of osteophytes at the dorsomedial surface of the talar neck (Fig. 3). The AMAO in the AMAI group had greater upper and inner bound protrusion distances than the pure CLAI group. There was no significant difference in the anterior bound protrusion distance of AMAO between the two groups (Table 2). There was no obvious AMAO in the AMAI group postoperatively (Fig. 2), and there were significant differences in AMAO protrusion distances between the two groups (Table 2). Because only one patient had a clear anteromedial anterior colliculus of medial malleolus osteophyte, the medial malleolus osteophyte was measured without statistical analysis. The proximity mapping technique was used to visualize the morphologic changes in the postoperative model compared to the preoperative model. The red and blue colors represented preoperative model deviations outside and inside the postoperative model (Fig. 4), and the AMAO was described as a polyhedral shape with a wide base and a narrow top.

**Table 2:** Quantification of the osteophytes

Variable		AMAI group (*n* = 29)	Pure CLAI group (*n* = 14)	*p* value
Upper- (mm)	Pre-	8.67 ± 2.39	3.62 ± 1.17	< 0.0001
	Post-	0.36 ± 0.12	3.62 ± 1.17	< 0.05
Inner- (mm)	Pre-	7.02 ± 2.86	2.05 ± 1.26	< 0.0001
	Post-	0.33 ± 0.27	2.05 ± 1.26	< 0.05
Anterior- (mm)	Pre-	1.98 ± 1.78	2.02 ± 1.54	n.s
	Post-	0.29 ± 0.27	2.02 ± 1.54	< 0.05

*Upper-* upper bound protrusion distance, *Inner-*, Inner bound protrusion distance, *Anterior-* Anterior bound protrusion distance, *AMAI* anteromedial ankle impingement, *CLAI* chronic lateral ankle instability, *Pre-* preoperatively, *Post-* postoperatively

“*p* value” indicated the comparison between the AMAI group and the pure CLAI group

## Discussion

The majority of ankle injuries can be treated conservatively. However, some patients with acute ankle joint injuries did not receive appropriate treatment, resulting in a repeated ankle sprain with lateral swelling and pain. The above symptoms are mainly driven by ligament relaxation caused by a lateral ligament tear, which easily leads to lateral ankle joint instability in the patient. Osteophytes form around the ankle when the joint is unstable for an extended period of time [21]. Walsh et al. performed arthroscopic treatment only for anterior ankle impingement and discovered that up to 84% had recurrence of osteophytes on imaging during the 5-year follow-up, which is thought to be associated with failure to restore ankle stability [6]. Yang et al., on the other hand, used ankle arthroscopy to stabilize the ankle joint with modified Broström repair of lateral ankle ligaments and to resect the anterior ankle osteophytes concurrently, and there was no osteophytes recurrence at a mean 3-year follow-up [26]. According to the research, lateral ankle ligament repair is critical in the treatment of CLAI. Our previous clinical work showed that AMAO was also found in some pure CLAI patients, all of them just accepted arthroscopic repair of ATFL without AMAO resection. No specific studies have been conducted to investigate the relationship between AMAO and AMAI in CLAI patients.

The normal ankle joint has about 20° of dorsiflexion and 50° of plantarflexion. The daily routine necessitates 10° of dorsiflexion and 20–25° of plantarflexion. Furthermore, stair climbing and physical exercises necessitate a greater range of dorsiflexion (20–30°) [27]. As we mentioned earlier, CLAI can cause ankle impingement, while AMAO can affect ankle dorsiflexion due to the obstruction of the ankle joint. The AMAO were the primary cause of decreased ankle dorsiflexion in the AMAI group in the current study. AMAO were resected under arthroscopic visualization until ankle dorsiflexion and inversion were achieved without impingement, and then, AFTL was repaired using the arthroscopic modified Broström technique [25]. Ankle arthroscopy has good efficacy, less injury, and fewer complications in the treatment of CLAI with AMAI. We found that arthroscopic treatment improved ankle dorsiflexion and alleviated anteromedial ankle pain in the AMAI group. Between preoperative and 2-year follow-up, there was a significant difference in ankle dorsiflexion in the AMAI group. We also concluded that AMAO resection procedures, as well as early joint mobility, are strongly related to the AOFAS score and patient satisfaction. Furthermore, at the 2-year follow-up, there was no significant difference in ankle dorsiflexion between the two groups.

Hence, the link between AMAO and AMAI in CLAI patients has significant implications for preoperative planning. Although the arthroscopic procedure is effective, the AMAO resection criterion is empirical, and the extent of osteophytes resection during the operation is determined by the surgeons. If the osteophytes are not adequately resected, they will still cause impingement, and excessive resection will result in secondary instability. As a result, preoperative quantitative and qualitative studies of the osteophytes appear to be especially important.

CT imaging can only see the three-dimensional shape of the ankle and cannot identify or quantify osteophytes. The morphology of osteophytes was successfully extracted and visualized in this study using intelligent software analysis. This is, to the best of our knowledge, the first study to visualize and quantify AMAO in CLAI patients with AMAI. We observed that the talar osteophytes were relatively fixed, concentrated within the midline sagittal plane of the talus, the talar neck, and the marginal region of the talar trochlear cartilage, which is consistent with previous reports. In the AMAI group, all patients had obvious osteophytes on the dorsomedial surface of the talar neck, but only one patient had obvious osteophytes on the anteromedial anterior colliculus of the medial malleolus. Therefore, we hypothesize that osteophytes on the dorsomedial surface of the talar neck are strongly linked to AMAI in CLAI patients. Notably, arthroscopic processing revealed that the large extent of the osteophytes on the dorsomedial surface of the talar neck would block the surgical field, necessitating their removal to expose the medial sulcus of the ankle mortise.

In the AMAI group, the osteophytes protruded 8.67 ± 2.39 mm from the upper border, 7.02 ± 2.86 mm from the inner border, and 1.98 ± 1.78 mm from the anterior border of the talar neck. Besides this, in the pure CLAI group, the osteophytes protruded 3.62 ± 1.17 mm from the upper border, 2.05 ± 1.26 mm from the inner border, and 2.02 ± 1.54 mm from the anterior border of the talar neck. The AMAO in the AMAI group had greater upper and inner bound protrusion distances than the pure CLAI group. The anterior bound protrusion distance of AMAO, on the other hand, did not differ significantly between the two groups. It was discovered that AMAO morphologic changes at the upper and inner bounds of the dorsomedial surface of the talar neck are significantly associated with AMAI in CLAI patients and should be strictly controlled during surgery as opposed to the anterior bound protrusion. There was no obvious AMAO in the AMAI group postoperatively, and all osteophyte protrusion distances were significantly lower than in the pure CLAI group. A 2-year follow-up revealed no significant difference in ankle dorsiflexion, AOFAS score, or VAS score between the two groups. Notably, ankle dorsiflexion and functional outcome scores in the pure CLAI group were only obtained after a 2-year follow-up, and more research is needed to determine whether the presence of osteophytes in the long term affects these results. In addition, in a follow-up study, we will investigate the threshold of each bound protrusion distance of the AMAO that causes AMAI in CLAI patients.

Our study has several limitations. To begin with, the sample size may have been insufficient to adequately assess osteophyte morphology. Second, the osteophytes on the dorsomedial surface of the talar neck form a three-dimensional uneven pattern, and the shape of the talus varies greatly from person to person, making standardized measuring difficult. Third, ankle impingement syndrome is a constantly changing condition. The current investigation was unable to imitate impingement with ankle dorsiflexion and plantarflexion in order to better examine osteophytes impingement. Fourth, the results of long-term postoperative follow-up were not included.

## Conclusion

CLAI with AMAI produces AMAO mostly on the dorsomedial surface of the talar neck, which has a polyhedral form with a wide base and a narrow top. AMAO’s upper and inner bound protrusion distances had a stronger link to impingement. In CLAI patients with AMAI, arthroscopic treatment of ATFL and AMAO yields satisfactory clinical effects.

## Data Availability

All data generated or analyzed during this study are included in this published article.

## Abbreviations

AMAO: Anteromedial ankle osteophytes
AMAI: Anteromedial ankle impingement
CLAI: Chronic lateral ankle instability
ATFL: Anterior talofibular ligament
AOFAS: American Orthopedic Foot and Ankle Society
VAS: Visual analog scale
CFL: Calcaneofibular ligament
PTFL: Posterior talofibular ligament
ALAI: Anterolateral ankle impingement
ADT: Anterior drawer test
MRI: Magnetic resonance imaging
DICOM: Digital Imaging and Communications in Medicine
ICP: Iterative closest point
